# Streamlining digital speech tests in Latin America and Africa

**DOI:** 10.1002/alz70863_110667

**Published:** 2025-12-23

**Authors:** Adolfo M Garcia, Franco Javier Ferrante, Gonzalo Nicolás Pérez, Alejandro Sosa Welford, Nicolás Pelella, Matías Caccia, Fernando Johann

**Affiliations:** ^1^ Cognitive Neuroscience Center (CNC), Universidad de San Andrés, Buenos Aires, Buenos Aires Argentina; ^2^ Global Brain Health Institute (GBHI), University of California San Francisco (UCSF); & Trinity College Dublin, Dublin Ireland; ^3^ Universidad de Santiago de Chile, Santiago, Santiago Chile; ^4^ Consejo Nacional de Investigaciones Científicas y Técnicas (CONICET), Buenos Aires Argentina; ^5^ Cognitive Neuroscience Center, University of San Andrés, Victoria, Buenos Aires Argentina; ^6^ Universidad de Buenos Aires, Buenos Aires Argentina; ^7^ Cognitive Neuroscience Centre, CABA, Buenos Aires Argentina; ^8^ School of Engineering, ORT University, Montevideo, Montevideo Uruguay; ^9^ Cognitive Neuroscience Center, CABA, Buenos Aires Argentina; ^10^ TELL Toolkit SA, Buenos Aires, Buenos Aires Argentina

## Abstract

**Background:**

Digital speech assessments are increasingly recognized as promising tools for identifying early indicators of cognitive decline and dementia. These solutions are cost‐effective, objective, and automated, offering potential to address global disparities in clinical evaluations. However, most available resources and findings originate from English‐speaking populations in high‐income countries, paradoxically fostering a new layer of inequity. To counter this scenario, in 2022 we launched the Toolkit to Examine Lifelike Language (TELL), a web‐based speech testing app focused on low‐ and middle‐income countries with distinct linguistic, cultural, technological, and translational needs. This presentation outlines the key adaptations and challenges faced during the localization of TELL to Latin American and African countries, focusing on our work in Chile and Kenya.

**Method:**

Interdisciplinary teams, involving researchers and clinicians from participating countries, were formed to refine back‐end and front‐end functionalities. Through iterative workshops, surveys, and interviews, country‐specific customizations were integrated into TELL's interface, prompts, data acquisition processes, preprocessing tools, metrics, and visualizations, culminating in TELL v.2.0.

**Results:**

We devised a protocol to translate app texts into Spanish and Kiswahili, using artificial intelligence and human review. Two pathways were established to select speech elicitation stimuli: a culture‐specific route (for pictorial content) and a culture‐neutral route (for video content). Software enhancements were made for data collection via videoconferencing and offline modes, accommodating local recruitment demands. A five‐step audio preprocessing system was devised to address varied acquisition environments, featuring automatic volume normalization, noise reduction, and speaker diarization. Metrics tailored to Spanish and Kiswahili were developed by integrating language‐agnostic (e.g., speech timing measures), language‐specific (e.g., psycholinguistic databases), cross‐linguistic (e.g., translation of online vocabularies), and multilingual (e.g., multilingual large language models) resources. Visualizations for clinical metrics were optimized based on expert feedback. Ongoing input from users highlights successes and areas for refinement.

**Conclusion:**

This experience expounds critical hurdles, solutions, and compromises involved in cross‐cultural adaptations of speech testing technologies. Lessons learned reveal good and perfectible practices for streamlining digital assessments across underserved world regions.

